# Toripalimab in combination with Anlotinib for unresectable hepatocellular carcinoma after SBRT: A prospective, single-arm, single-center clinical study

**DOI:** 10.3389/fonc.2023.1113389

**Published:** 2023-03-17

**Authors:** Yongbiao Chen, Hanyin Hong, Wenzheng Fang, Xia Zhang, Huachun Luo, Zhijian Chen, Jianda Yu, Weiqiang Fan, Xiaobin Chi, Yonghai Peng

**Affiliations:** ^1^ Department of Hepatobiliary Surgery, 900 Hospital of the Joint Logistics Support Force, Fuzhou, China; ^2^ Fuzong Clinical Medical College, Fujian Medical University, Fuzhou, China; ^3^ Department of Oncology, 900 Hospital of the Joint Logistics Support Force, Fuzhou, China; ^4^ Department of Hepatobiliary Disease, 900 Hospital of the Joint Logistics Support Force, Fuzhou, China; ^5^ Department of Radiology, 900 Hospital of the Joint Logistics Support Force, Fuzhou, China; ^6^ Medical Oncology of Cangshan Hospital Area, 900 Hospital of the Joint Logistics Support Force, Fuzhou, China

**Keywords:** Toripalimab, Anlotinib, stereotactic body radiotherapy (SBRT), unresectable hepatocellular carcinoma (uHCC), clinical trial

## Abstract

**Objective:**

Exposing tumor antigens to the immune system is the key to ensuring the efficacy of immunotherapy. SBRT is the main way to reveal the specifical antigens of tumors which can enhance the immune response. We aimed to explore the clinical efficacy and safety of Toripalimab combined with Anlotinib for uHCC after SBRT.

**Methods:**

This is a prospective, single-arm, explorative clinical study. uHCC patients with an ECOG PS score of 0–1, Child–Pugh class A or B, and BCLC stage B or C were included and treated with SBRT(8Gy*3) followed by 6-cycle combinational therapy with Toripalimab and Anlotinib. The primary endpoint was progression-free survival (PFS) and the secondary endpoints were objective response rate (ORR), disease control rate (DCR), overall survival (OS), and incidence of treatment-related adverse events (TRAEs). Continuous variables were presented as medians and ranges. Survivals were studied with the Kaplan-Meier method. Categorical data were expressed as n (percentage).

**Results:**

Between June 2020 and October 2022, a total of 20 patients with intermediate-advanced uHCC were enrolled. All cases had multiple intrahepatic metastases, or macrovascular invasion, or both, among whom 5 cases with lymph node or distant metastases. Until September 2022, the median follow-up time was 7.2 months (range, 1.1-27.7 months). Median survival time could not be assessed at the moment, based on iRecist, median PFS was 7.4 months (range, 1.1-27.7 months), ORR 15.0%, and DCR 50.0%. 14 patients experienced treatment-related adverse events with an incidence of 70%. The overall survival rates at 18 months and 24 months were 61.1% and 50.9%, respectively. And the progression-free survival rates were 39.3% and 19.7%.

**Conclusion:**

Exposure of specific antigens of HCC *via* SBRT may improve the efficacy of combinational therapy with Toripalimab and Anlotinib for uHCC with manageable adverse effects, which deserves further exploration.

**Clinical trial registration:**

www.clinicaltrials.gov, identifier ChiCTR2000032533.

## Introduction

Hepatocellular carcinoma is a prevalent malignant tumor of the digestive system and is one of the leading causes of cancer-related deaths. According to global cancer statistics for 2020, it ranks 6th in incidence and 3rd in tumor-related mortality worldwide, and the situation in China is even worse (1, 2). Less than 10% of people with hepatocellular carcinoma survive the disease for five years. The disease develops quietly and around half of the patients are already in a middle to late stage when they are first diagnosed. Macrovascular invasion is seen on imaging, and the prognosis is exceedingly dismal, with an expected survival of about 2 to 5 months (3, 4).

Currently, multi-target tyrosine kinase inhibitors (TKIs) such as sorafenib, Lenvatinib, and Donafenib as first-line targeted therapeutic agents for advanced hepatocellular carcinoma have been shown to slow tumor progression and extend survival time. However, the ORR of sorafenib is only 2%, and Lenvatinib was non-inferior to sorafenib in terms of OS (13.6 vs. 12.3 months) in REFLECT clinical trial. Among them, Lenvatinib had the highest ORR of 24.1% (5–7). The primary study endpoint of survival time was not met with Nivolumab in phase III clinical trial CheckMate-459 (8). Similarly, KEYNOTE-240, a phase III study of Pembrolizumab for second-line treatment of advanced hepatocellular carcinoma, failed to demonstrate superiority of anti-PD-1 therapy in comparison with routine therapy (9). Though monotherapy of Immune checkpoint inhibitors (ICIs) obtains higher ORR compared to Sorafenib, but the clinical efficacy and survival outcomes of patients with uHCC are still unsatisfied.

At present, ICIs in combination with anti-angiogenic therapies are currently a popular direction in the systemic therapy of intermediate-advanced uHCC. In the IMBrave 150 phase III clinical trial, Atezolizumab combined with Bevacizumab outperformed the sorafenib group in uHCC patients in terms of OS and PFS (10). It was recommended by the FDA as a first-line treatment for untreated advanced hepatocellular carcinoma. Several clinical studies such as Pembrolizumab plus Lenvatinib, Sintilimab plus a bevacizumab biosimilar (IBI305), Camrelizumab plus Apatinib, have shown longer OS and PFS as well as higher ORR (11–13). Transcatheter artery chemoembolization (TACE) is one of the preferred locoregional treatments which is recommended by the guidelines for patients with uHCC (14). Necrosis of tumor tissue induced by TACE can release tumor antigens, which may promote tumor-specific immune responses (15). The synergistic anti-tumor effect of TACE combined with PD - (L) 1 inhibitor and TKI has been confirmed that the combinational therapy can significantly improve the survival and oncological benefit of Chinese patients compared with TACE monotherapy. However, TACE-induced embolic syndrome increases patient suffering and abnormal liver function are often seen in patients with intermediate to advanced HCC, which trigger us to think it over and seek for other effective local treatment options. The high-level radiobiologic effect of high-dose irradiation by stereotactic body radiotherapy (SBRT), which is significantly superior to conventional fractionated radiotherapy, can induce reactive oxygen species-related DNA damage, cause immunogenic death of tumor cells (16–18). Tumor cell death exposes a large number of tumor-specific antigens, which activate cross-presentation of antigen-presenting cells to effector T cells, evoking local immune responses and also inducing adaptive immune responses outside the radiation area and distant metastases to produce a “distant effect” (19). Meanwhile, it is now preferred that anti-VEGF plays a facilitative and synergistic role when patients receive ICIs.

The combination of multiple regimens has shown superior therapeutic effects and will be a popular research direction. We believe that after exposing the specific antigens of tumor cells *via* SBRT may lead to better efficacy of immunotherapy. The purpose of this article is to summarize the effectiveness and safety of the combination treatment regimen of Toripalimab combined with Anlotinib after SBRT in patients with unresectable hepatocellular carcinoma.

## Materials and methods

### Patients

The diagnosis of HCC was based on histological examination of tumor tissue obtained by percutaneous needle biopsy or clinical diagnostic criteria for hepatocellular carcinoma staging proposed in China are as follows: 1) two radiological imaging assessments with typical features of hepatocellular carcinoma (arterial phase early enhancement and portal vein phase early washout); 2) one imaging assessment showing typical features of hepatocellular carcinoma with abnormal serum AFP levels (>400ng/mL). The Japanese Hepatocellular Carcinoma Study Group defined unresectable hepatocellular carcinoma(uHCC) as a double tumor with extensive invasion of the liver due to a large single or multiple tumor or invasion of major vessels including the main portal vein (Vp4) and the inferior vena cava (Vv3).

Inclusion criteria: (1) age from18 to 75; ECOG PS score: 0-1; Anticipated survival of more than 3 months; (2) Child-Pugh class A; (3) Hepatocellular carcinoma that meets the Chinese Diagnostic Criteria for Primary Liver Cancer (2022) or is diagnosed by pathology; (4) BCLC stage B or C, intermediate and advanced stages that are not suitable for surgical operation and/or disease progression after local treatment.

Exclusion criteria: (1) previous TKIs, ICIs, or TKIs in combination with ICIs; (2) Anticipated survival of less than 3 months; (3) Active period of hepatitis B and C (including both symptoms and blood indicators. Symptoms include fatigue, anorexia, deepening of urine color, pain over the liver region, jaundice, etc; Blood indicators include elevated transaminases, bilirubin and HBV-DNA copies ≥500copies/mL); (4) combined with other tumors.

The following information should be completed within 4 weeks before the therapy starts: basic information, tumor diagnosis, history of treatment, underlying diseases, ECOG PS score, vital signs, physical examination, radiological images (contrast-enhanced CT or MRI, PET-CT or ECT when necessary), ECG, blood routine, urine routine, stool routine, blood biochemistry, coagulation function test, thyroid function test, AFP, HIV/HBV/HCV virus test.

The trial has been registered at www.clinicaltrials.gov (ClinicalTrials.gov identifier: ChiCTR2000032533). The study was approved by China Ethics Committee of Registering Clinical Trials (Ethical review document number: ChiECRCT20200128) and informed consent was obtained from all patients, whose data will be used for research purposes, according to the Declaration of Helsinki.

### Procedures

**Figure 1:**
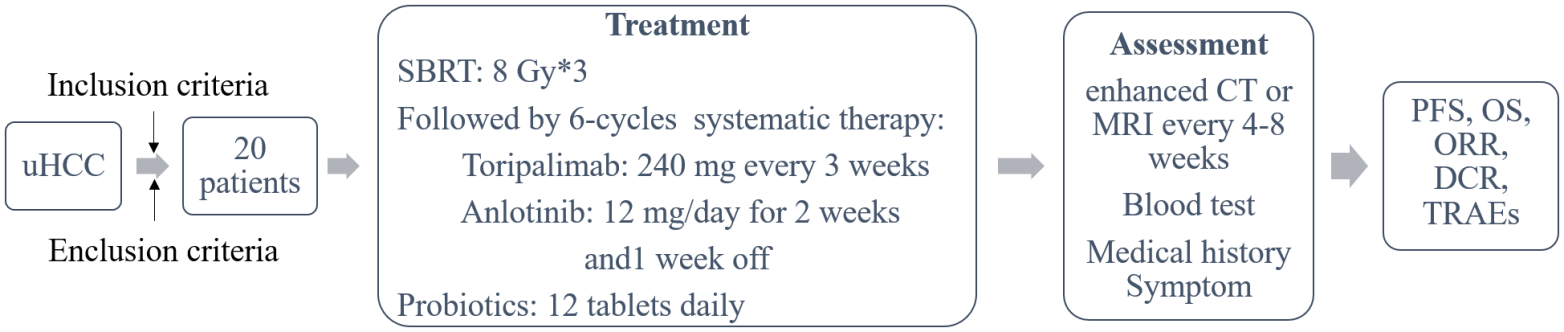
Flowchart of the study.

Targeted area was localized under CT guidance and then the patients received SBRT at a total dose of 24 Gy completed in the next 3 days. Toripalimab 240 mg intravenously every 3 weeks was initiated after the final SBRT fraction with one-day interval. Meanwhile, all patients received Anlotinib 12 mg/day for 2 weeks with one-week off. Treatment continued until clinical or radiographic disease progression, dose-limiting toxicity, withdrawal from study, or death. During the therapeutic period, patients take 12 tablets of Live Combined Bifidobacterium, Lactobacillus and Enterococcus Capsules daily to maintain the balance of gut microbiome (**Figure 1**).

### Response and toxicity evaluation

The primary study endpoint was progression-free survival (PFS) in unresectable hepatocellular carcinoma treated with combined theraputic regimen of Toripalimab plus Anlotinib after SBRT. Secondary study endpoints are objective response rate (ORR), disease control rate (DCR), overall survival (OS), and incidence of treat-related adverse events. According to RECIST 1.1 criteria, tumor response was assessed by enhanced CT or MRI every 4-8 weeks as complete response (CR), partial response (PR), stable disease (SD), or progressive disease (PD). The ORR was defined as the proportion of patients with the best response (either CR or PR) ≥4 weeks after the criteria for response were first met, while the DCR was defined as CR, PR, and SD. Progress-free survival (PFS) was calculated from the initiation of SBRT until the date when the criteria for PR or CR were first met. Pathologic CR and major pathologic response were defined as the complete absence and less than or equal to 10% of viable tumor cells in the resected specimen, respectively. Treatment-related adverse events during the patient’s treatment were followed up and recorded according to the Common Terminology Criteria for Adverse Events Version 5.0.

### Follow-up

Evaluation of study end points began from the start date of SBRT. Computed tomography scans were scheduled after every 3-4 cycle of Toripalimab, or earlier if clinical diagonosis of relapse. Adverse events and treatment responses were monitored by oncologists and surgeons. When patients have completed 6 cycles of treatment, later treatments were decided by a multidisciplinary team after discussion and the patient’s wishes. The follow-up deadline was October 1, 2022.

### Statistical analysis

Continuous variables were presented as medians and ranges. Survivals were studied with the Kaplan-Meier method. Categorical data were expressed as n (percentage). All statistical analyses were performed using Statistical Package for Social Sciences (SPSS) software (Version 25, SPSS, Inc., Chicago, IL, USA). Graphs were plotted by GraphPad Prism 8.0.1.

## Results

### Patient characteristics

Up to July 2022, a total of 20 patients were enrolled in this clinical trial. One patient died of upper gastrointestinal hemorrhage 33 days after treatment. The median duration of follow-up was 7.2 months. Of 20 patients (median age 57 years, range 38-73 years, 19 males and 1 female), 19 cases (95.0%) were infected with HBV. 12 cases (60.0%) had baseline AFP ≥ 400 ng/mL. 16 cases (80.0%) were accompanied with macrovascular invasion and 6 cases (30.0%) with extrahepatic metastases (1 left adrenal metastasis, 1 greater omentum metastases, 2 lymph node metastases, and 2 pulmonary metastases). The general information about the patients and the treatment details are shown in **Tables 1** and **2**.

**Table 1:** Patient demographics and baseline characteristics.

Characteristics	Patients (n, %)
Gender	
Male	19 (95.0)
Female	1 (5.0)
Age, years, n(%)	
<65	15 (75.0)
≥65	5 (25.0)
ECOG PS, n (%)	
0	14 (70.0)
1	6 (30.0)
Hepatitis B infection	19 (95.0)
Pre-treatment AFP, n (%)	
< 400 ng/mL	8 (40.0)
≥ 400 ng/mL	12 (60.0)
Tumor number, n (%)	
Solitary	5 (25.0)
Multiple	15 (75.0)
Macrovascular invasion and thrombus formation	16 (80.0)
Extrahepatic metastasis, n (%)	6 (30)
adrenal metastasis	1 (5.0)
greater omentum metastases	1 (5.0)
lymph node metastases	2 (10.0)
pulmonary metastases	2 (10.0)
BCLC staging, n (%)	
B	3 (15.0)
C	17 (85.0)

**Table 2:** Treatment details and outcome of patients.

No	Age	Gender	ECOG	BCLC	AFP	Tumor number	Macrovascular invasion	Extrahepatic metastasis	Pre-treatment	Treatment cycles	Tumor response	statue
1	42	male	0	C	17.31	Solitary	no	adrenal	hepatectomy	5	SD	alive
2	72	male	0	B	6.26	Solitary	no	no	no	5	SD	alive
3	42	male	1	C	normal	Multiple	no	lung	TACE	3	PD	death
4	59	male	0	C	970.8	Multiple	PVTT	non	no	3	PD	death
5	63	male	0	C	961.4	Solitary	PVTT	lung	no	1	SD	alive
6	43	male	0	C	996.6	Multiple	PVTT/IVCTT	lymph node	no	12	SD	death
7	57	male	1	C	212.3	Multiple	PVTT	no	hepatectomy	3	PD	alive
									TACE			
8	51	male	0	C	49823	Multiple	PVTT/HVTT	lymph node	no	6	PR	alive
9	54	male	0	C	>121000	Multiple	PVTT	no	no	5	PR	alive
10	56	male	1	C	>121000	Multiple	PVTT	no	no	1	PD	death
11	38	male	0	B	41302	Solitary	no	no	no	6	PR	alive
12	54	male	0	B	386.9	Multiple	PVTT/SMVT	no	no	2	PD	alive
13	51	male	1	C	22396	Multiple	PVTT	no	no	1	PD	death
14	67	male	1	C	175	Solitary	PVTT	no	no	2	PD	death
15	72	female	1	C	23.89	Multiple	PVTT	no	no	6	PD	alive
16	64	male	1	C	1873	Multiple	PVTT/HVTT	no	no	3	PD	alive
17	45	male	0	C	>121000	Multiple	PVT	no	no	6	SD	alive
18	63	male	2	C	698.4	Multiple	PVTT/IVCTT	no	no	3	PD	death
19	72	male	0	C	217.5	Multiple	HVTT	no	no	1	SD	alive
20	70	male	0	C	48656	Multiple	PVTT	omentum	no	1	SD	alive

### Tumor response and safety

**Figure 2:**
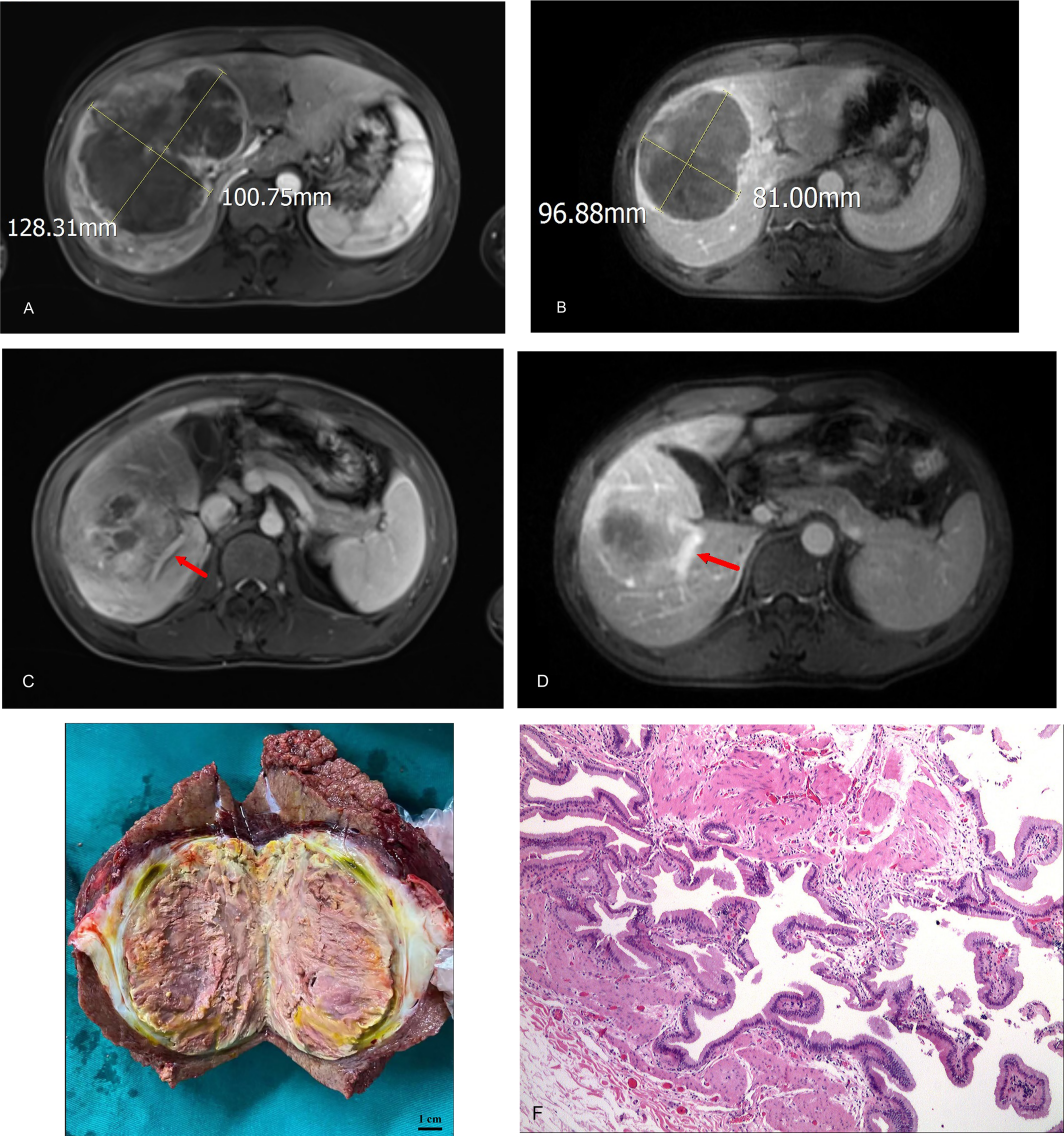
**(A)** initial tumor size (portal vein phase); **(B)** post-treatment/pre-operative tumor size (portal vein phase); **(C)** portal vein involvement(pre-treatment); **(D)** portal vein involvement (post-treatment/pre-operative); **(E)** Tumor specimens; **(F)** Pathological images (200X).

**Figure 3:**
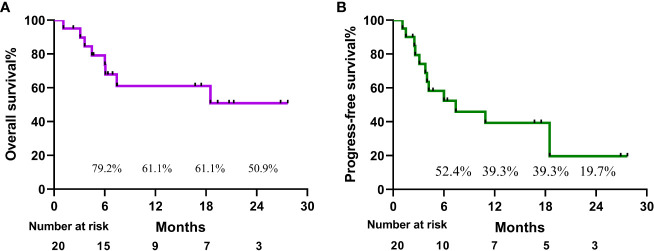
Kaplan-Meier curves of overall survival **(A)** and progression-free survival **(B)**.

A patient with BCLC stage B whose preoperative enhanced MRI indicated a huge occupation in the middle liver lobe with involvement of the right portal vein and the right hepatic vein was initially diagnosed with hepatocellular carcinoma. After 6 cycles of therapies, the tumor responded well and shrank significantly, and level of AFP decreased from 410,302 ng/ml to 13.9 ng/ml. After the joint consultation of the multi-disciplinary treatment, it was decided to perform mid-liver tumor resection and cholecystectomy. Postoperative pathology showed a large area of tumor cell necrosis and a small amount of tumor cell remnants which was considered as pathological complete response (pCR), 2 patients reached partial response (PR) (**Figure 2**). 7 cases had stable disease (SD), and 9 cases showed progressive disease (PD) (**Table 3**). The median disease-free survival was 7.4 months, and the median overall survival could not be assessed at the moment (**Figure 3**). ORR and DCR was 15.0%, 50.0% respectively.

**Table 3:** Tumor responses assessment (iRECIST).

Best response	BCLC stage	
	BCLC B No. (%) Overall (n = 3)	BCLC C No. (%) Overall (n =17)
Complete response	1 (33.3)	0 (0)
Partial response	1 (33.3)	1 (5.9)
Stable disease	1 (33.3)	6 (35.3)
Progressive disease	0 (0)	10 (58.8)
Objective response rate, n (%)	2 (66.7)	1 (5.9)
Disease control rate, n (%)	3 (100)	7 (41.2)

14 patients (70.0%) experienced TRAEs at any grade. The most common TRAEs were elevated alanine aminotransferase, elevated aspartate aminotransferase, elevated blood bilirubin, decreased white/neutrophil/lymphocytes, fatigue, hypertension, decreased appetite, and gastrointestinal symptoms (abdominal pain, abdominal distension, diarrhea, etc.) (**Table 4**). 2 patients had grade 3 TRAEs (diarrhea≥7/day, elevated bilirubin>5ULN). One patient with portal hypertension and esophageal and gastric varices died from acute upper gastrointestinal hemorrhage which was defined as 5-grade TRAE. During the therapeutic procedure, most TRAEs were evaluated as mild and manageable.

**Table 4:** Treatment related adverse events.

Adverse Event	Any Grade (n=14)	Grade 1-2 (n=11)	Grade 3-4 (n=2)	Grade 5 (n=1)
Evaluated transaminase	7(35.0%)	7(35.0%)	0	0
Elevated blood bilirubin	8(40.0%)	7(35.0%)	1(5.0%)	0
Leukopenia/Neutropenia/lymphocytopenia	6(30.0%)	6(30.0%)	0	0
Anemia	2(10.0%)	2(10.0%)	0	0
Thrombocytopenia	2(10.0%)	2(10.0%)	0	0
Fatigue	6(30.0%)	6(30.0%)	0	0
Decreased appetite	4(20.0%)	4(20.0%)	0	0
Gastrointestinal symptoms	5(25.0%)	4(20.0%)	1(5.0%)	0
Hypertension	3(15.0%)	3(15.0%)	0	0
Hand-foot syndrome	2(10.0%)	2(10.0%)	0	0
Gastrointestinal hemorrhage	1(5.0%)	0	0	1(5.0%)

## Discussion

As far as we know, there are no many reports about SBRT followed by ICIs and TKIs in intermediate and advanced HCC. Both TACE and SBRT can directly kill tumor cells to reduce tumor burden. SBRT can trigger not only local immune responses but also adaptive immune responses outside the radiation area and distant metastases, which is called “distant effect”. A single dose of 8 Gy*3 irradiation regimens has been proven to have a better distant effect and improve the OS and PFS in the NSCLC patients (20). Meta-analysis (21) of 1580 Chinese patients with advanced hepatocellular carcinoma showed that in patients with advanced hepatocellular carcinoma combined with portal vein or inferior vena cava trunk thrombosis, TACE plus radiotherapy prolonged survival compared with TACE alone or TACE plus sorafenib, indicating that advanced hepatocellular carcinoma combined with thrombosis is sensitive to radiotherapy. Chiang et al. (22) reported 5cases with uHCC received SBRT then followed by Nivolumab, achieving 100% ORR, which demonstrated impressive tumor control from the combination of SBRT and checkpoint inhibitors. Studies have certified that SBRT or conventional radiotherapy combined with PD-1, either before or during treatment, could provide better survival outcomes than patients who do not (23). A retrospective analysis of a small sample showed that palliative radiotherapy combined with PD-1/PD-L1 and anti-angiogenic agents was safe and effective in patients with BCLC C with an ORR of 40% and prolonged survival time (24).

Compelling reports suggest that VEGF has immunosuppressive properties for tumors apart from activating angiogenesis-related processes. In the past, anti-VEGF antibodies were thought to act more favorably to chemotherapy, for example, by normalizing tumor vasculature, but nowadays their facilitative and synergistic role in immunotherapy is preferred. Strong correlation was found between the increase of tumor infiltrating lymphocytes (such as CD4+and CD8+T cells) and the normalization of blood vessels imposed by VEGF pathway inhibitors (25, 26). All above convince us that SBRT together with anti-angiogenic will Significantly enhance the efficacy of immunotherapy to achieve better anti-tumor effects.

Notably, patients were supplemented with sufficient doses of enteric probiotics to maintain the balance of gut microbiome which should be given adequate attention. Existing views suggest that immune responses triggered by microbial-related molecular patterns such as intestinal flora translocation and ecological dysbiosis with intestinal flora metabolism are the main mechanisms by which intestinal flora promote the development of hepatocellular carcinoma through the intestine-hepatic axis (27). Numerous studies have shown that regulating gut microbiome may be a safer, low-cost potential strategy for the prevention or treatment of liver cancer by means of improving the immunological response to ICIs and enhancing the anti-tumor effects (28, 29), and plays an important role in improving the prognosis of patients with intermediate-advanced HCC.

Numerous clinical trials have shown that combinational therapy with PD-1 inhibitor and TKI achieved remarkable effect, with higher tumor response and longer survival than monotherapy. Combination regimens of multiple treatments can complement the shortcomings of a single treatment and provide better outcomes. However, the side effects caused by the combinational therapies will increase the risk of treatment-related adverse events and the safety needs further investigation. In our cohort, all patients in our study showed good tolerance to our regimen except one patient died of acute upper gastrointestinal hemorrhage. Several randomized clinical trials suggested that patients treated with PD-1 inhibitor and TKI were able achieved PFS ranging from 4.6 to 8.6 months approximately. A majority of the cases included had a combination of vascular carcinoma thrombosis (80%) and intrahepatic metastases (75%) with advanced stage. Based on this premise, we achieved a PFS of 7.4 months, with a 2-year overall-survival rate and a progression-free survival rate of 59.9% and 29.7%, respectively.

The limitations of this study conclude as follows: small sample size, single-center, as well as the selection bias of cases during the epidemic of COVID-19. In addition, ICIs and TKIs are still expensive at present and patients need good financial support. Also, the follow-up period was short in some cases, and the different treatment doses due to different treatment cycles may influence the assessment of efficacy. All of the above may affect the scientific validity of our conclusion of this trial.

In conclusion, exposure of tumor antigens of HCC *via* SBRT may improve the efficacy of combinational therapy with Toripalimab and Anlotinib for uHCC with manageable adverse effects, which deserves further exploration.

## Data availability statement

The raw data supporting the conclusions of this article will be made available by the authors, without undue reservation.

## Ethics statement

The studies involving human participants were reviewed and approved by China Ethics Committee of Registering Clinical Trials. The patients/participants provided their written informed consent to participate in this study.

## Author contributions

Conception and design: YP, YC, XC, HH. (II) Provision of study materials or patients: YC, YP, WF, XZ, HL, WF. (III)Collection and assembly of data: HH, ZC, JY. (IV) Data analysis and interpretation: HH, YC, ZC, JY. (V) Manuscript writing: HH, YC. (VI) Final approval of manuscript: All authors. All authors contributed to the article and approved the submitted version.
